# Obesity Is a Positive Modulator of IL-6R and IL-6 Expression in the Subcutaneous Adipose Tissue: Significance for Metabolic Inflammation

**DOI:** 10.1371/journal.pone.0133494

**Published:** 2015-07-22

**Authors:** Sardar Sindhu, Reeby Thomas, Puthiyaveetil Shihab, Devarajan Sriraman, Kazem Behbehani, Rasheed Ahmad

**Affiliations:** 1 Immunology and Innovative Cell Therapy Unit, Dasman Diabetes Institute (DDI), P.O. Box 1180, Dasman, Kuwait; 2 Tissue Bank Core Facility, Dasman Diabetes Institute (DDI), P.O. Box 1180, Dasman, Kuwait; University of Leicester, UNITED KINGDOM

## Abstract

The role of IL-6R/IL-6 axis in metabolic inflammation remains controversial. We determined the changes in adipose tissue expression of IL-6R and IL-6 in obese, overweight, and lean non-diabetic individuals. Subcutaneous adipose tissue biopsies were collected from 33 obese, 22 overweight, and 10 lean individuals and the expression of IL-6R, IL-6, TNF-α, MCP-1, IP-10, CD11b, CD163, and CD68 was detected by immunohistochemistry; results were also confirmed by real-time RT-PCR and confocal microscopy. The data were compared using unpaired t-test and the dependence between two variables was assessed by Pearson’s correlation test. Obese individuals showed higher IL-6R expression (103.8±4.807) in the adipose tissue as compared with lean/overweight (68.06±4.179) subjects (*P*<0.0001). The elevated IL-6R expression correlated positively with body mass index (BMI) (r=0.80 *P*<0.0001) and percent body fat (r=0.69 *P*=0.003). The increased IL-6R expression in obesity was also confirmed by RT-PCR (Obese: 3.921±0.712 fold; Lean/Overweight: 2.191±0.445 fold; *P*=0.0453) and confocal microscopy. IL-6 expression was also enhanced in obese adipose tissue (127.0±15.91) as compared with lean/overweight (86.69±5.25) individuals (*P*=0.03) which correlated positively with BMI (r=0.58 *P*=0.008). IL-6 mRNA expression was concordantly higher in obese (16.60±2.214 fold) versus lean/overweight (9.376±1.656 fold) individuals (*P*=0.0108). These changes in the IL-6R/IL-6 expression correlated positively with the adipose tissue expression of CD11b (IL-6R r=0.44 *P*=0.063; IL-6 r=0.77 *P*<0.0001), CD163 (IL-6R r=0.45 *P*=0.045; IL-6 r=0.55 *P*=0.013), TNF-α (IL-6R r=0.73 *P*=0.0003; IL-6 r=0.60 *P*=0.008), MCP-1 (IL-6R r=0.61 *P*=0.005; IL-6 r=0.63 *P*=0.004) and IP-10 (IL-6R r=0.41 *P*=0.08; IL-6 r=0.50 *P*=0.026). It was, therefore, concluded that obesity was a positive modulator of IL-6R and IL-6 expression in the adipose tissue which might be a contributory mechanism to induce metabolic inflammation.

## Introduction

With the increasing use of energy-rich foods and overnutrition worldwide, the incidence of obesity has risen to epidemic proportions and the risks for obesity-associated diseases are also escalating in all age groups [1]. The serious health problems that are associated with obesity include type-2 diabetes, cardiovascular disease and certain types of hormonally-related cancers. The white adipose tissue is not only a site for energy storage but is also an active endocrine organ that secretes over 50 cyto-/chemokines and bioactive mediators called adipokines which are involved in lipid metabolism, insulin sensitivity, immunity, angiogenesis, and inflammation [2,3]. Obesity is marked by a state of chronic low-grade inflammation called metabolic inflammation or meta-inflammation in which the immune cells, especially monocytes, are activated, infiltrate the expanding adipose tissue and become differentiated as resident adipose tissue macrophages (ATMs). ATMs are classified by the expression of M1 or proinflammatory markers (IL-6, TNF-α, MCP-1, CD11c, iNOS), M2 or anti-inflammatory markers (CD163, CD206, Arginase-1), and common markers (CD11b, CD68) [4].

Interleukin (IL)-6 is an important cytokine which is secreted by macrophages, adipocytes, and other sources including skeletal muscle, fibroblasts, and endothelial cells [5,6]. It is a systemic regulator of body weight and lipid metabolism [7,8]. It is also associated with obesity and insulin resistance; however it is unclear whether it plays a harmful [9–11] or a protective role [12,13] in this regard. Therefore, we determined the obesity-related changes in the adipose tissue expression of IL-6 receptor (IL-6R) and IL-6 and assessed their relationship with signature inflammatory mediators or markers in this compartment. Herein, we present the data showing that obesity enhances gene and protein expression of the IL-6R and IL-6 in the human subcutaneous adipose tissue which correlates positively with the local expression of several inflammatory markers.

## Materials and Methods

### Study population

This study included 65 non-diabetic adults that were divided into 10 lean (3 males and 7 females aged 28–55 yrs; BMI = 22.549±2.264 kg/m^2^), 22 overweight (14 males and 8 females aged 29–71 yrs; BMI = 27.882±1.574 kg/m^2^), and 33 obese individuals (16 males and 17 females aged 24–66 yrs; BMI = 34.985±3.233 kg/m^2^). The three groups were comparable with regard to fasting blood glucose, and glycated hemoglobin levels. Regarding comorbidities, one lean and 3 obese individuals had hyperlipidemia while one obese individual, each, had coronary artery disease, lung disease and osteoporosis. The clinico-demographic data of the study subjects are summarized in Table 1. All participants gave written informed consent and the study was approved by the Ethical Review Committee of Dasman Diabetes Institute, Kuwait.

**Table 1:** Clinico-demographic data of study participants.

Parameter	Lean	Overweight	Obese
**Male (N)**	3	14	16
**Female (N)**	7	8	17
**Age (Yrs.)**	28–55	29–71	24–66
**Body mass index (BMI) (kg/m** ^**2**^ **)**	22.549 ± 2.264	27.882 ± 1.574	34.985 ± 3.233
**High-density lipoproteins (mmol/L)**	1.623 ± 0.417	1.235 ± 0.297	1.197 ± 0.311
**Low-density lipoproteins (mmol/L)**	3.39 ± 0.929	3.163 ± 0.666	3.303 ± 0.992
**Triglycerides (mmol/L)**	0.69 ± 0.224	1.181 ± 0.612	1.306 ± 0.830
**Fasting blood glucose (mmol/L)**	4.943 ± 0.639	5.52 ± 1.459	5.352 ± 0.732
**Cholesterol (mmol/L)**	5.344 ± 1.132	4.921 ± 0.718	5.079 ± 1.101
**Glycated hemoglobin (HbA1c) %**	5.52 ± 0.346	5.838 ± 1.538	5.691 ± 0.621
**Co-morbidities**			
**Hyperlipidemia (N)**	1	0	3
**Coronary artery disease (N)**	0	0	1
**Lung diseases (N)**	0	0	1
**Osteoporosis (N)**	0	0	1

### Anthropometric and physio-clinical measurements

Anthropometric and physical measurements included body weight, height, waist circumference as well as systolic and diastolic blood pressure. Height and weight were measured with barefoot participants wearing light indoor clothing using calibrated portable electronic weighing scales and portable inflexible height measuring bars; the waist circumference at the highest point of the iliac crest and the mid-axillary line was measured using constant tension tape at the end of a normal expiration with arms relaxed at the sides. The waist-to-hip ratios were calculated, and the whole-body composition including percentage of body fat (PBF), soft lean mass and total body water were measured by the use of IOI 353 Body Composition Analyzer (Jawon Medical, South Korea). Blood pressure was measured by using Omron HEM-907XL digital automatic sphygmomanometer (Omron Healthcare Inc. IL, USA). An average of the 3 blood pressure readings, with 5–10 min rest between each, was obtained. BMI was calculated using standard BMI formula i.e. body weight (kg)/ height (m^2^).

Regarding clinical laboratory measurements, peripheral blood was collected by phlebotomist through venipuncture from overnight-fasted (minimum 10 hrs) individuals and samples were analyzed for fasting blood glucose, glycated hemoglobin (HbA1c), fasting insulin, and lipid profile. Glucose and lipid profiles were measured using Siemens dimension RXL chemistry analyzer (Diamond Diagnostics, Holliston, MA, USA) and HbA1c was measured by using Variant device (BioRad, Hercules, CA, USA). All assays were carried out following instructions as recommended by the manufacturers.

### Collection of subcutaneous adipose tissue samples

Human adipose tissue samples (~0.5g) were collected via abdominal subcutaneous fat pad biopsy lateral to the umbilicus using standard surgical method. Briefly, the periumbilical area was sterilized by alcohol swabbing and then locally anesthetized using 2% lidocaine (2ml). Through a small superficial skin incision (0.5cm), the fat tissue was collected. After removal, biopsy tissue was further incised into small pieces, rinsed in cold phosphate buffered saline (PBS), fixed in 4% paraformaldehyde for 24 hr and then embedded in paraffin for further use. At the same time, freshly collected adipose tissue samples (~50–100 mg) were preserved in RNAlater or embedded in optimal cutting temperature (OCT) and stored at -80°C until use.

### Antibodies

The following rabbit polyclonal primary antibodies (abcam, USA) were used: anti-IL6R antibody (ab128008; 1:8000 dilution), anti-IL6 antibody (ab154367; 1:400 dilution), anti-TNFα antibody (ab9635; 1:800 dilution), anti-MCP1 antibody (ab9669; 1:400 dilution), anti-IP10 antibody (ab9807; 1:400 dilution), anti-CD11b antibody (ab52478; 1:600 dilution), anti-CD163 antibody (ab87099; 1:1000 dilution), and anti-CD68 antibody (ab125157; 1:200 dilution). Mouse anti-rabbit monoclonal antibody conjugated with horse radish peroxidase (HRP) polymer chain was used as secondary antibody (EnVision Kit from Dako, Glostrup, Denmark).

### Immunohistochemistry (IHC)

Paraffin-embedded sections (4μm thick) of subcutaneous adipose tissue were deparaffinized in xylene and rehydrated through descending grades of ethanol (100%, 95%, and 75%) to water. Antigen retrieval was performed by placing slides in target retrieval solution (pH6.0; Dako, Glostrup, Denmark) under pressure cooker boiling for 8 min and cooling for 15 min. After washing in PBS, endogenous peroxidase activity was blocked with 3% H_2_O_2_ for 30 min and non-specific antibody binding was clocked with 5% nonfat milk for 1hr and 1% bovine serum albumin (BSA) solution for 1hr. Slides were treated overnight with primary antibodies at room temperature using dilutions as recommended by manufacturers. After washing with PBS (0.5% Tween), slides were incubated for 1hr with secondary antibody conjugated with HRP polymer chain and color was developed using 3,3ʹ-diaminobenzidine chromogen substrate. Specimens were washed in running tap water, lightly counterstained with Harris hematoxylin, dehydrated through ascending grades of ethanol (75%, 95%, and 100%), cleared in xylene, and finally mounted in dibutyl phthalate xylene (DPX).

For analysis, digital photomicrographs of the entire adipose tissue sections (20×; Olympus BX51 Microscope, Japan) were used to quantify the immunohistochemical staining in three different regions to assess the regional heterogeneity in tissue samples and the regions were outlined using Aperio ImageScope software (Aperio Vista, CA, USA). Aperio positive pixel count algorithm (version 9) was used to quantify the amount of specific staining in the region. The number of positive pixels was normalized to the number of total (positive and negative) pixels to account for variations in size of the region sampled. Color and intensity thresholds were established to detect immunostaining as positive and background staining as negative pixels. Once the conditions were established, all slides were analyzed using the same parameters. The resulting color markup of analysis was confirmed for each slide.

### Real-time reverse-transcription polymerase chain reaction (RT-PCR)

Total cellular RNA was purified using RNeasy kit (Qiagen, Valencia, CA, USA) as per manufacturer’s instructions. Briefly, the adipose tissue samples in RNAlater or OCT-embedded were thawed and homogenized in Qiazol lysis solution (Qiagen, Valencia, CA, USA) using TissueRuptor (Qiagen, Hildon, Germany) at 33,000rpm for 40 sec. The homogenate was treated with chloroform and separated into aqueous and organic phases by centrifugation at 12,000 ×g for 15 min at 4°C. The upper aqueous RNA phase was collected, 70% ethanol was added, and the sample was applied to an RNeasy spin column to allow total RNA binding with the membrane and to wash out phenol and other contaminants. High-quality RNA was then eluted in RNase-free water. The quantity of the isolated RNA was determined using Epoch Spectrophotometer System (BioTek, Winooski, USA) and the quality was assessed by formaldehyde-agarose gel electrophoresis. The RNA samples (1μg each) were reverse transcribed to yield cDNA using random hexamer primers and TaqMan reverse transcription reagents (High Capacity cDNA Reverse Transcription kit; Applied Biosystems, CA, USA).

For real-time RT-PCR, cDNA (50ng) was amplified using TaqMan Gene Expression MasterMix (Applied Biosystems, CA, USA) and gene-specific 20× TaqMan Gene Expression Assays as follows: (IL-6R) Hs01075666; (IL-6) Hs00985639_m1; and (GAPDH) Hs03929097_g1 (Applied Biosystems, CA, USA) containing forward and reverse primers and a target-specific TaqMan minor groove binder (MGB) probe labeled with 6-fluorescein amidite (FAM) dye at the 5' end and non-fluorescent quencher (NFQ)-MGB at the 3' end of the probe, for 40 cycles of PCR reaction using a 7500 Fast Real-Time PCR System (Applied Biosystems, CA, USA). Each cycle consisted of denaturation for 15 sec at 95°C, annealing/extension for 1 min at 60°C which started after uracil DNA glycosylase (UDG) activation (50°C for 2 min) and AmpliTaq Gold enzyme activation (95°C for 10 min). The amplified glyceraldehyde 3-phosphate dehydrogenase (GAPDH) expression was used as internal control to normalize the differences in individual samples and gene expression levels of IL-6R and IL-6 relative to controls (lean adipose tissue) were calculated using -2^ΔΔCt^ method. Relative mRNA expression was expressed as fold expression over average of control gene expression. The expression level in control samples was assumed as 1 and data were presented as mean ± SEM values.

### Statistical analysis

The data obtained were expressed as mean ± SEM values; group means were compared using unpaired *t*-test and the linear dependence between two variables was assessed by determining Pearson’s correlation coefficient ‘r’ values. GraphPad Prism software (version 6.05; San Diego, CA, USA) was used for statistical analysis and graphical representation of the data. All *P*-values ≤0.05 were considered statistical significant.

## Results

### Adipose tissue expression of IL-6R is enhanced in obesity

**Fig 1:**
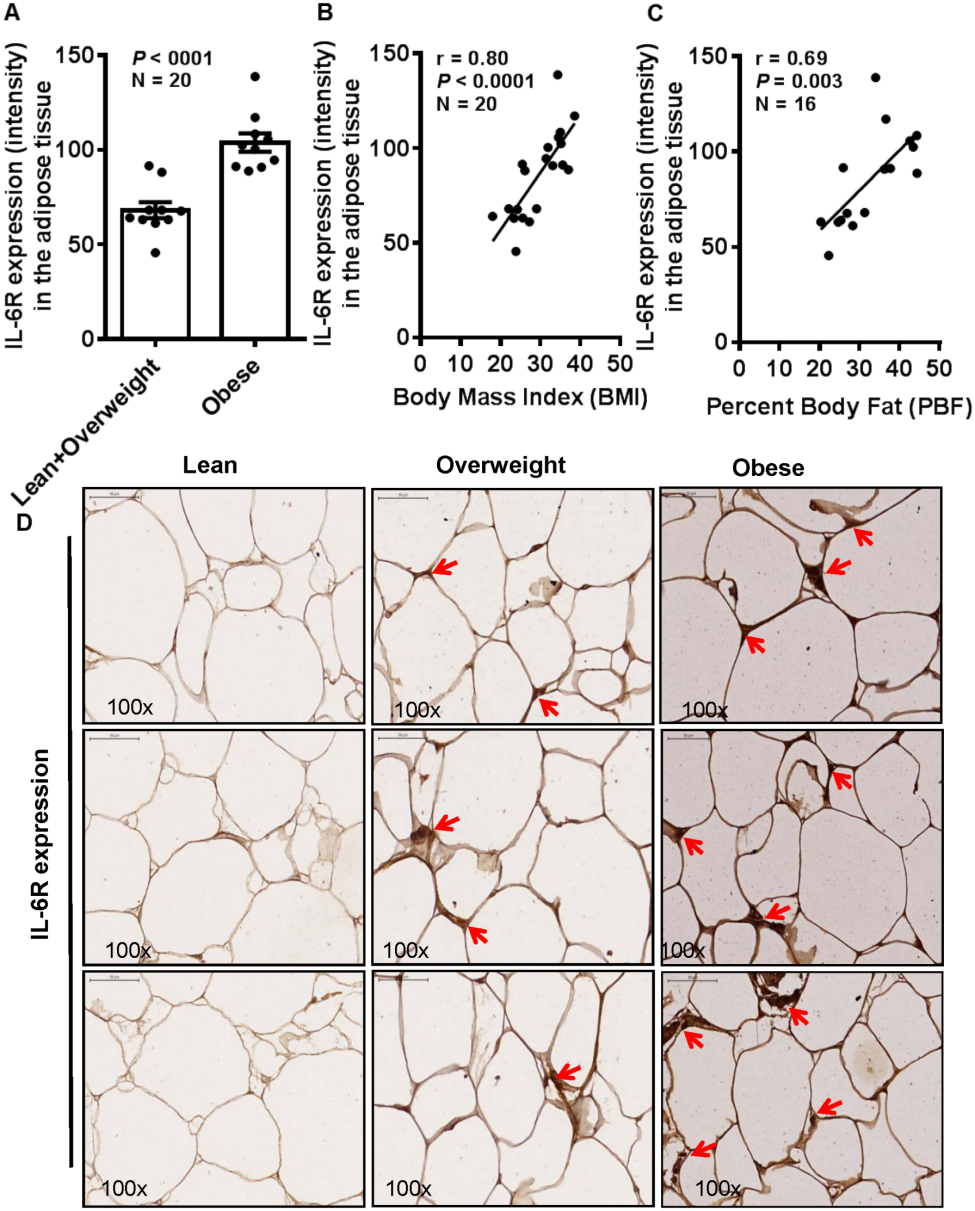
Adipose tissue expression of IL-6R is enhanced in obesity. The subcutaneous adipose tissue samples were obtained by surgical biopsy from 10 lean/ overweight (BMI = 20.285 to 29.456) and 10 obese (BMI = 31.752 to 38.218) non-diabetic individuals and protein expression (intensity) of IL-6R was measured by immunohistochemistry (IHC). Group means were compared using unpaired *t*-test and all *P*-values ≤0.05 were considered statistically significant. The data show that (**A**) IL-6R expression was significantly elevated in the adipose tissue samples from obese (103.8±4.807) as compared with lean/ overweight (68.06±4.179) individuals (*P*<0.0001). (**B**) The increase in IL-6R expression correlated positively with body mass index (BMI) (r = 0.80, *P*<0.0001) and (**C**) percent body fat (PBF) (r = 0.69, *P* = 0.002). (**D**) The representative IHC photomicrographs (100× magnification) of IL-6R staining intensity (arrows) in the adipose tissue samples from lean, overweight, and obese individuals, 3 each, are shown. Similar photomicrographs at a wider field view (20× magnification) are shown in supporting S1 Fig.

**Fig 2:**
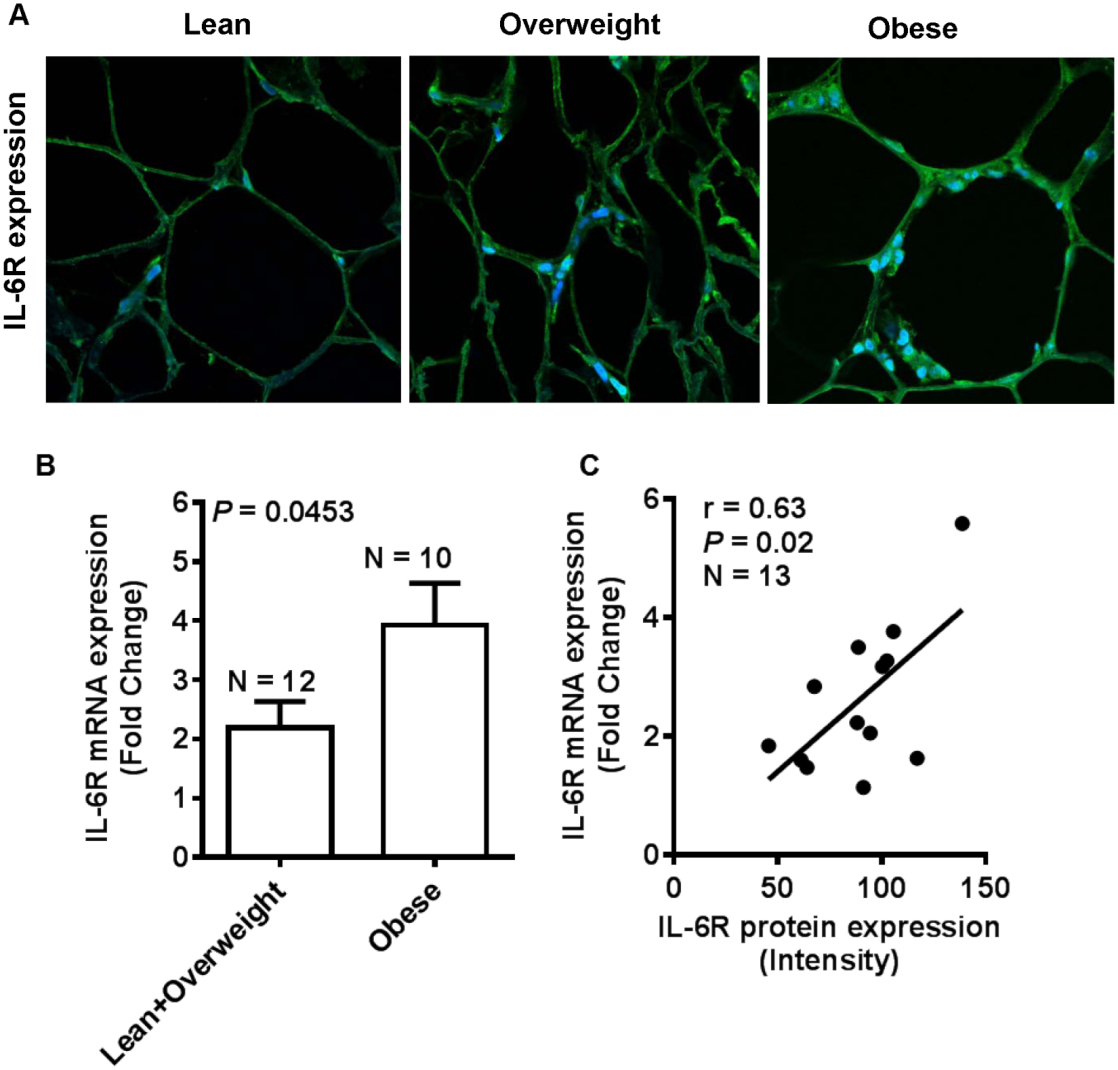
IL-6R expression changes are validated by confocal microscopy and real time RT-PCR. (**A**) The increased IL-6R protein expression in the adipose tissue samples of obese and overweight individuals as compared with lean individuals was also confirmed by confocal microscopy. (**B**) Real-time RT-PCR was used to assess the IL-6R gene expression in the adipose tissue samples from lean, overweight, and obese individuals. The data obtained confirmed the significantly increased IL-6R gene expression in obese adipose tissues (3.921±0.712 fold) as compared with lean/ overweight (2.191±0.445 fold) adipose tissue samples (*P* = 0.0453). (**C**) A strong positive correlation was found between IL-6R gene and protein expression in the adipose tissues (r = 0.63, *P* = 0.02).

The emerging evidence supports adipose tissue as an endocrine, paracrine, autocrine and juxtacrine organ. During obesity, adipocytes enlarge and the adipose tissue undergoes cellular and molecular changes affecting the systemic metabolism and immune responses. IL-6 is an important adipokine and the changes in cognate receptor expression on target tissues are critical to immune reactivity to this cytokine. We asked whether obesity modulated the IL-6R expression in the adipose tissue. To this end, our data show that IL-6R protein expression was significantly enhanced in the subcutaneous adipose tissues from obese individuals (103.8±4.807) as compared with lean and overweight (68.06±4.179) individuals (*P*<0.0001) (Fig 1A). Furthermore, this increase in IL-6R expression correlated positively with clinical markers of obesity including BMI (r = 0.80, *P*<0.0001) (Fig 1B) and percent body fat (PBF) (r = 0.69, *P* = 0.003) (Fig 1C). The representative IHC photomicrographs of three individuals in each category show the markedly enhanced IL-6R expression in obese as compared with overweight and lean adipose tissues (Fig 1D). The IHC data were further confirmed by confocal microscopy which also showed similar results (Fig 2A). Also, to see whether the changes in IL-6R protein expression were corroborated at the gene expression level, we used real-time RT-PCR and, as expected, we found that IL-6R mRNA expression was significantly elevated in obese individuals (3.921±0.712 fold) as compared with lean and overweight (2.191±0.445 fold) subjects (*P* = 0.0453) (Fig 2B). A good agreement was found between IL-6R gene and protein expression (r = 0.63, *P* = 0.02) (Fig 2C).

### IL-6 expression is enhanced concordantly in the obese adipose tissue

**Fig 3:**
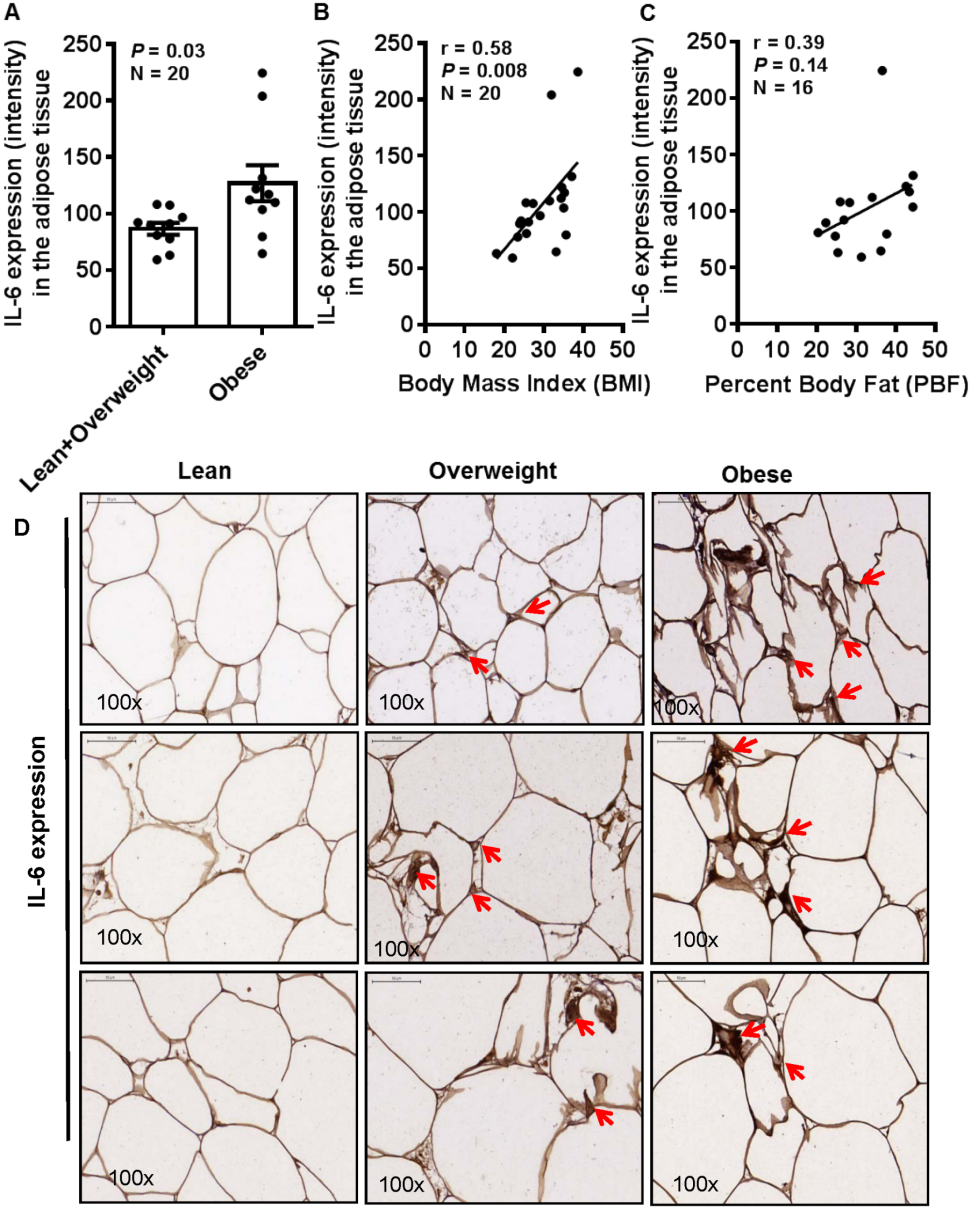
IL-6 expression is also elevated in the obese adipose tissues. (**A**) Adipose tissue expression of the IL-6R ligand i.e. IL-6 expression was also determined by immunohistochemistry (IHC) and was found to be significantly elevated in the adipose tissues from obese (127.0±15.91) as compared with overweight and lean (86.69±5.25) individuals (*P* = 0.03). (**B**) The increase in IL-6 expression correlated positively with body mass index (BMI) (r = 0.58, *P* = 0.008); (**C**) while the correlation with percent body fat (PBF) was found to be non-significant (r = 0.39, *P* = 0.14). (**D**) The representative IHC photomicrographs (100× magnification) of IL-6 staining intensity (arrows) in the adipose tissue samples from lean, overweight, and obese individuals, 3 each, are shown. Similar photomicrographs at a wider field view (20× magnification) are shown in supporting S2 Fig.

**Fig 4:**
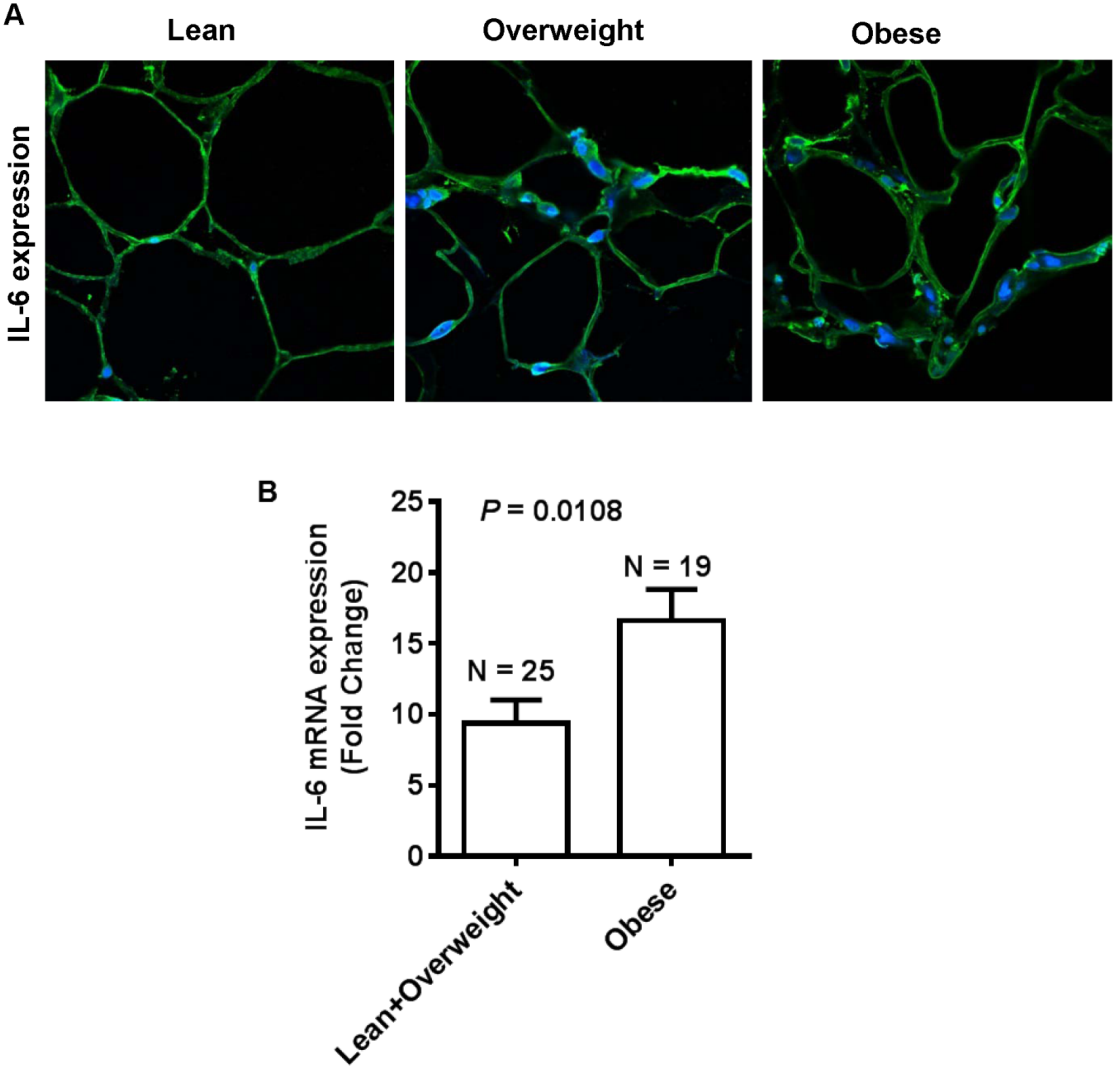
IL-6 expression changes are confirmed by confocal microscopy and real time RT-PCR. (**A**) The increased IL-6 protein expression in the adipose tissues of obese and overweight individuals as compared with lean subjects was confirmed by confocal microscopy. (**B**) Real-time RT-PCR data also confirmed the increased IL-6 gene expression in obese adipose tissues (16.60±2.214 fold) as compared with lean/ overweight (9.376±1.656 fold) adipose tissue samples (*P* = 0.0108).

IL-6 may exert both the inflammatory and anti-inflammatory responses in the adipose tissue. We further asked whether the enhanced IL-6R expression in the obese adipose tissue was paralleled by increased expression of IL-6 in this compartment. To this effect, our data show that IL-6 protein expression in the adipose tissue was significantly increased in obese individuals (127.0±15.91) as compared with lean and overweight (86.69±5.25) counterparts (*P* = 0.03) (Fig 3A). The increased expression of IL-6 correlated positively with BMI (r = 0.58, *P* = 0.008) (Fig 3B); however, the correlation with PBF was found to be non-significant (r = 0.39, *P* = 0.14) (Fig 3C). The representative IHC photomicrographs from three individuals in each group show markedly enhanced IL-6 protein expression in the obese as compared with overweight and lean adipose tissue (Fig 3D). The IHC data were further validated by confocal microscopy which also corroborated elevated IL-6 expression in the obese adipose tissue (Fig 4A). As expected, IL-6R mRNA expression was also significantly upregulated in obese individuals (16.60±2.214 fold) as compared with lean and overweight (9.376±1.656 fold) subjects (*P* = 0.0108) (Fig 4B).

### Enhanced IL-6R and IL-6 expression correlates with macrophage markers expression

**Fig 5:**
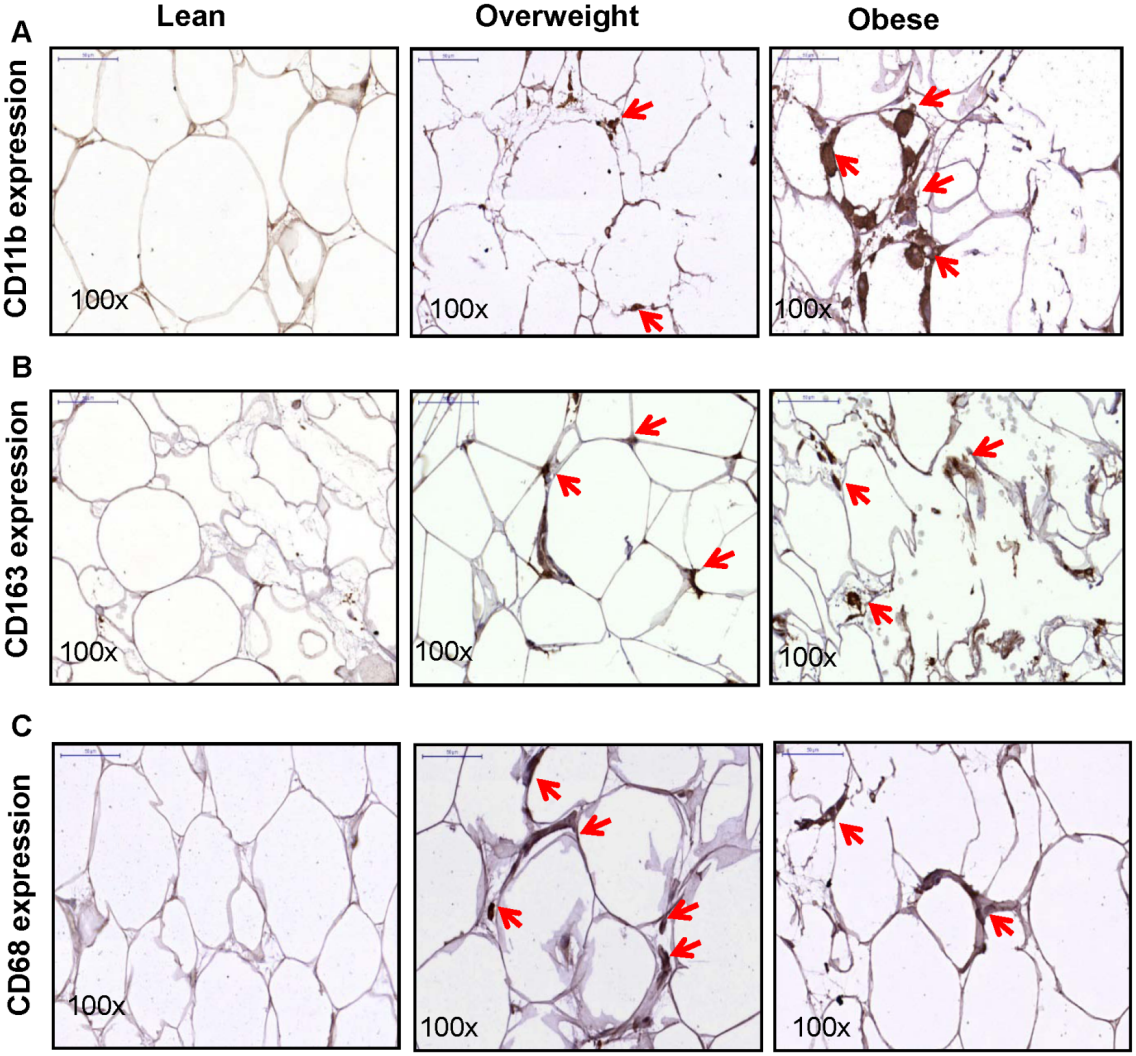
Enhanced expression of monocytes/ macrophage markers in the obese adipose tissue. The protein expression (intensity) of monocyte/ macrophage markers was detected by immunohistochemistry (IHC) in the adipose tissue samples from lean, overweigh, and obese individuals, 10 each. As shown by representative IHC photomicrographs (100× magnification), expression of (**A**) CD11b, (**B**) CD163, and (**C**) CD68 was found to be markedly elevated in overweight and obese adipose tissue samples as compared with lean samples.

**Fig 6:**
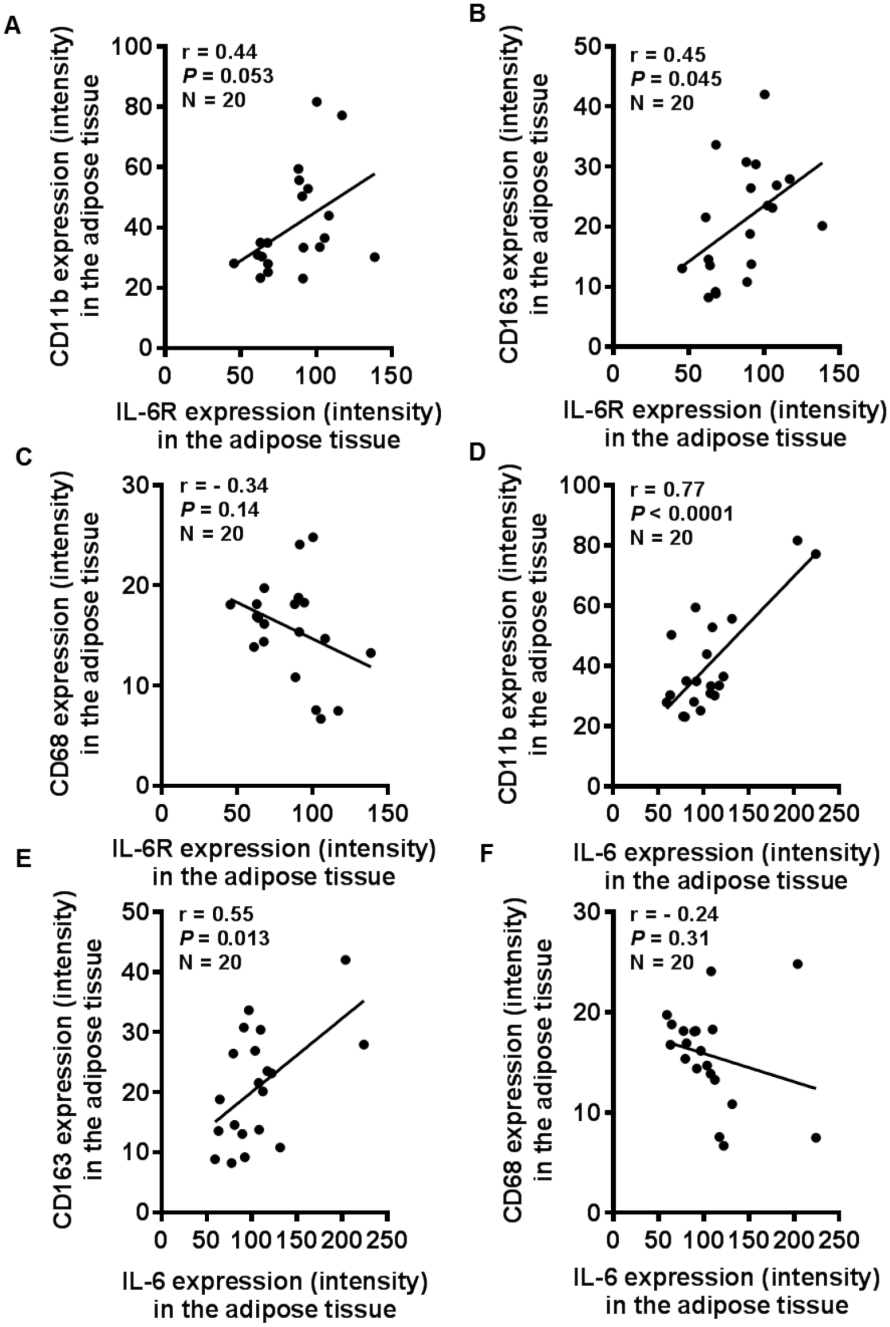
Adipose tissue IL-6R/IL-6 expression relates with the local expression of monocyte/ macrophage markers. The immunohistochemistry (IHC) data show a positive correlation of IL-6R with (**A**) CD11b (r = 0.44, *P* = 0.053) and (**B**) CD163 (r = 0.45, *P* = 0.045); and also of IL-6 with (**D**) CD11b (r = 0.77, *P*<0.0001) and (**E**) CD163 (r = 0.55, *P* = 0.013). However, the correlations of CD68 with (**C**) IL-6R (r = -0.34, *P* = 0.14) and (**F**) IL-6 were found to be non-significant (r = -0.24, *P* = 0.31).

We next asked whether the enhanced adipose tissue expression of IL-6R and IL-6 in obesity was related to the tissue inflammatory state represented by ATM colonization. In this regard, adipose tissue expression of monocyte/ macrophage markers CD11b (Fig 5A) and CD163 (Fig 5B) was found to be significantly upregulated in overweight and obese individuals as compared with lean while the CD68 expression (Fig 5C) was relatively enhanced in overweight/ obese as compared with lean subjects. The adipose tissue IL-6R expression correlated positively with that of CD11b (r = 0.44, *P* = 0.053) (Fig 6A) and CD163 (r = 0.45, *P* = 0.045) (Fig 6B) while its negative correlation with CD68 was found to be non-significant (r = -0.34, *P* = 0.14) (Fig 6C). Similarly, the adipose tissue expression of IL-6 correlated positively with CD11b (r = 0.77, *P*<0.0001) (Fig 6D) and CD163 (r = 0.55, *P* = 0.013) (Fig 6E) and the negative correlation with CD68 was found to be non-significant (r = -0.24, *P* = 0.31) (Fig 6F). The gene expression analysis yielded similar results (data not shown).

### IL-6R and IL-6 expression correlates with adipose tissue expression of signature inflammatory mediators

**Fig 7:**
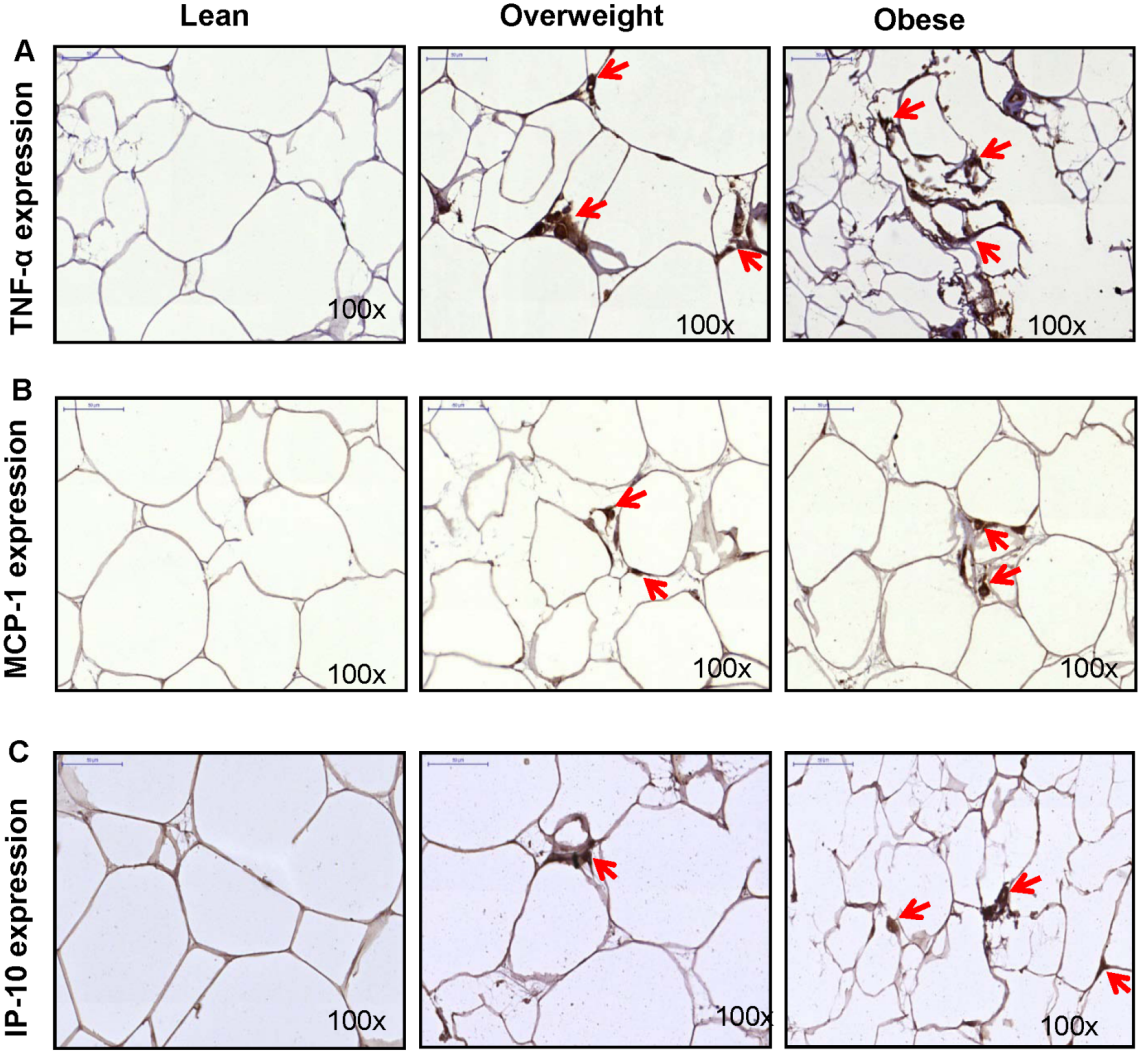
Adipose tissue IL-6R/IL-6 expression correlates with the local expression of signature inflammatory mediators. The protein expression (intensity shown by arrows) of TNF-α, MCP-1, and IP-10 was detected in the adipose tissue samples from lean, overweigh, and obese individuals by immunohistochemistry (IHC). As revealed by representative (IHC) photomicrographs (100× magnification), expression of (**A**) TNF-α, (**B**) MCP-1, and (**C**) IP-10 was found to be markedly increased in overweight and obese adipose tissue samples as compared with lean tissue samples.

**Fig 8:**
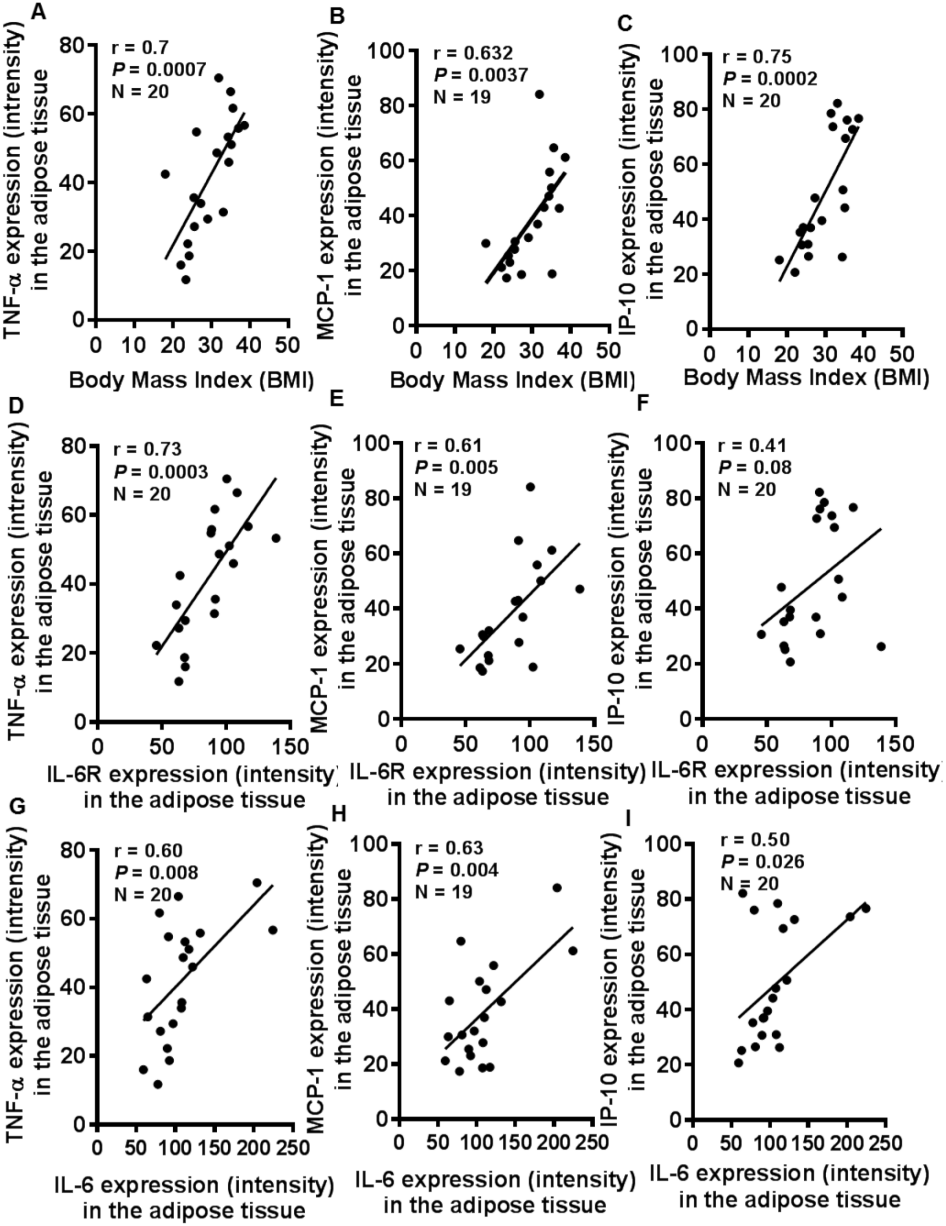
Adipose tissue expression of the signature inflammatory mediators correlates with body mass index (BMI) as well as with IL-6R/IL-6 expression. The adipose tissue expression of TNF-α, MCP-1, IP-10, IL-6R, and IL-6 was assessed by immunohistochemistry (IHC). The IHC data show a positive correlation of body mass index (BMI) with (**A**) TNF-α (r = 0.70, *P* = 0.0007), (**B**) MCP-1 (r = 0.63, *P* = 0.004), and (**C**) IP-10 (r = 0.75, *P* = 0.0002). Besides, the adipose tissue expression of IL-6R related directly with local expression of (**D**) TNF-α (r = 0.73, *P* = 0.0003), (**E**) MCP-1 (r = 0.61, *P* = 0.005), and (**F**) IP-10 (r = 0.41, *P* = 0.08). Similarly, IL-6 expression also correlated with the adipose tissue expression of (**G**) TNF-α (r = 0.60, *P* = 0.008), (**H**) MCP-1 (r = 0.63, *P* = 0.004), and (**I**) IP-10 (r = 0.50, *P* = 0.026).

TNF-α is a signature proinflammatory cytokine and in the adipose tissue, it can be produced by adipocytes and macrophages. MCP-1 or CCL-2 is a CC-chemokine secreted by monocytes/ macrophages, dendritic cells, and adipocytes. IP-10 or CXCL-10 is a CXC-chemokine secreted by monocytes/ macrophages, fibroblasts and endothelial cells. Next, we asked whether the IL-6R/IL-6 expression was concordant with tissue expression of these inflammatory mediators. To this end, we found that the adipose tissue TNF-α (Fig 7A), MCP-1 (Fig 7B), and IP-10 (Fig 7C) were elevated in overweight and obese as compared with lean individuals. BMI correlated positively with TNF-α (r = 0.70, *P* = 0.0007) (Fig 8A), MCP-1 (r = 0.63, *P* = 0.0037) (Fig 8B), and IP-10 (r = 0.75, *P* = 0.0002) (Fig 8C). Next, the increased IL-6R expression also correlated with TNF-α (r = 0.73, *P* = 0.003) (Fig 8D), MCP-1 (r = 0.61, *P* = 0.005) (Fig 8E), and IP-10 (r = 0.41, *P* = 0.08) (Fig 8F). Similarly, the IL-6 expression also correlated with TNF-α (r = 0.60, *P* = 0.008) (Fig 8G), MCP-1 (r = 0.63, *P* = 0.004) (Fig 8H), and IP-10 (r = 0.50, *P* = 0.026) (Fig 8I). Similar data were obtained by gene expression analysis (data not shown).

## Discussion

The role of IL-6 in obesity-associated chronic low-grade metabolic inflammation is controversial. It is also unclear how the adipose tissue expression of IL-6R and IL-6 is modulated by obesity. First, our data show the elevated adipose tissue expression of IL-6R and IL-6 in obese individuals and the changes correlate with clinical indicators of obesity including BMI and PBF. The IHC images show that IL-6R and IL-6 expression was largely confined to crown like structures formed by monocytes/ macrophages in the periphery of degenerating adipocytes. The elevated adipose tissue expression of IL-6R and IL-6 was also confirmed by confocal microscopy and real-time RT-PCR. The gene and protein expression were found to be mutually congruent. Our data showing IL-6 perturbations in obesity extend and confirm the previous findings [8,14]; while the data on the adipose tissue IL-6R changes in obesity add further information.

IL-6R is a heterodimer of IL-6 binding unit called IL-6Rα/gp80 and IL-6 signal transducer called IL-6Rβ/gp130 [15]. The IL-6Rα/IL-6 complex associates with gp130 which dimerizes and initiates intracellular signaling called *cis*-signaling which generates anti-inflammatory responses. The shedding of IL-6R by metalloproteases yields the soluble isoform (sIL-6R) which can bind to IL-6 to form immune complexes (IL-6/sIL-6R) [16–18], and IL-6 binds to IL-6R or sIL-6R with comparable affinity [19]. Notably, IL-6 cannot directly bind to gp130 whereas the IL-6/sIL-6R immune complexes can, which implies that gp130-expressing cells, and most cells express gp130 [20], can be stimulated by this immune complex even in the absence of IL-6R via a *trans*-signaling mechanism which induces a proinflammatory response. Thus, *trans*-signaling by enlarging the spectrum of IL-6 target cells may exacerbate inflammatory responses; and may also induce the inflammatory CD4^+^T_H_17 polarization [21]. Conversely, the presence of sIL-6R/sgp130 immune complex in the circulation may have a buffering effect through the formation of IL-6/sIL-6R/sgp130 trimeric complex as only the remaining IL-6 left unbound to this complex will be available to engage in a biological response.

Adipose tissue is an important source of circulating IL-6 and the expanding adipose tissue in obesity may contribute high levels of IL-6 in the circulation. It was separately reported by two different studies that plasma [14] and adipose tissue [22] levels of IL-6 correlated better than TNF-α with obesity and insulin resistance. Of note, IL-6 is also regarded as a myokine and in skeletal muscle, it acts as an energy sensor by activating AMP-activated protein (AMPK) kinase and increasing glucose disposal, fat oxidation, and lipolysis [23]. IL-6 produced in skeletal muscle is released in the circulation after prolonged physical activity which may exert anti-obesity effect on liver and adipose tissue via glucose homeostasis and exercise-induced lipolysis. As an anti-inflammatory cytokine, IL-6 was reported to inhibit the effects of proinflammatory TNF-α [24], promote M2 macrophage polarization, and improve insulin resistance [12]. IL-6 signaling was also associated with muscle growth and myogenesis; however, it also caused atrophy and muscle wasting [25]–an effect which is supported by another study showing that IL-6 acts on both protein synthesis and degradation [26]. On the other hand, IL-6 treatment of mice blunted the insulin-stimulated insulin receptor substrate (IRS)-2-associated phosphatidylinositol 3-kinase (PI3K) activity in liver and also reduced insulin-stimulated glucose uptake in skeletal muscle via defective IRS-1-associated PI3K signaling and elevated fatty acyl-CoA levels [27]. Overall, the role of IL-6 in insulin sensitivity and glucose homeostasis remains controversial [28]; and we speculate that the obesity-associated perturbations in IL-6 and its receptor likely have diverse effects in different tissues and organs.

Our data show the enhanced adipose tissue expression of CD11b and CD163 macrophage markers in overweight and obese as compared with lean individuals. IL-6R and IL-6 expression correlated positively with CD11b and CD163 markers in the adipose tissue. Increased CD11b and CD163 expression reflects the enhanced chemotaxis and tissue infiltration by inflammatory monocytes/ macrophages as previously reported [29,30]. Both adipocytes and macrophages produce proinflammatory mediators that lead to low-grade chronic inflammation via autocrine/ paracrine mechanisms. CD11b is a pan-macrophage marker related with adhesion/chemotaxis, phagocytosis, and is upregulated on activated cells. CD163 is a scavenger receptor expressed by monocytes and macrophages during differentiation or immune activation [31–33]. Our data did not show an increase in CD68 marker in obesity. Of note, anti-CD68 antibodies were previously reported to cross react with a glycoprotein present in primary granules of neutrophils, phagosomes and lysosomes [34]. Since it correlated negatively with IL-6 and other inflammatory mediators or markers, it might be possible that the tissue inflammatory state promoted proteolytic shedding of CD68. Consistent with this argument, soluble CD68 was reported to be present in serum and urine samples [35]. Overall, CD163 is considered to be a more specific marker for cells of monocytic/ macrophage lineage. Herein, the combined detection of three markers was relevant to confirm the monocytic infiltration and macrophage colonization in the adipose tissue. We also found that TNF-α, MCP-1, and IP-10 were upregulated in obese adipose tissue samples which correlated with increased IL-6R and IL-6 expression. Our data are corroborated, in part, by the previous findings showing elevated TNF-α levels in obesity [36,37] and macrophage recruitment and angiogenesis in breast adipose tissue [38]. TNF-α is a signature proinflammatory cytokine that promotes the expression of other inflammatory cyto-/chemokines and induces insulin resistance by inhibiting IRS-1 signaling pathway [39]. MCP-1 and IP-10 are both induced in the adipose tissue during inflammation in obesity and may cause insulin resistance [40–42]. Although different cells can produce MCP-1, adipocytes are the most important source and it was reported to cause adipose tissue inflammation even in the absence of macrophages and other leukocytes [43].

Nonetheless, this study involves a few caveats as well. First, the sample size is limited. Second, this study elucidates the obesity-related changes in IL-6R/IL-6 together with other inflammatory mediators/ markers. Therefore, further studies will be required to validate and extend these findings by including: (i) larger sample sizes; (ii) more inflammatory markers; and (iii) changes in both tissue and circulatory levels.

In conclusion, our data support a model in which human obesity leads to the elevated expression of IL-6R and IL-6 in the adipose tissue, with increased tissue expression of TNF-α, MCP-1, IP-10 and infiltration by CD11b^+^/CD163^+^ macrophages as the underlying features of meta-inflammation.

## Supporting Information

S1 Fig Enhanced IL-6R expression in the subcutaneous adipose tissue in obesity. The subcutaneous adipose tissue samples were obtained by surgical biopsy from 10 lean/ overweigh (BMI = 20.285 to 29.456) and 10 obese (BMI = 31.752 to 38.218) non-diabetic individuals and protein expression (intensity) of IL-6R was measured by immunohistochemistry (IHC). The representative IHC photomicrographs (20× magnification) of IL-6R staining intensity (arrows) in the adipose tissue samples from lean, overweight, and obese individuals, 3 each, are shown.
(TIFF)

S2 Fig Enhanced IL-6 expression in the subcutaneous adipose tissue in obesity. The subcutaneous adipose tissue samples were obtained by surgical biopsy from 10 lean/ overweigh (BMI = 20.285 to 29.456) and 10 obese (BMI = 31.752 to 38.218) non-diabetic individuals and protein expression (intensity) of IL-6 was measured by immunohistochemistry (IHC). The representative IHC photomicrographs (20× magnification) of IL-6 staining intensity (arrows) in the adipose tissue samples from lean, overweight, and obese individuals, 3 each, are shown.
(TIFF)
