# Supplementary figures and images for: Obesity Is a Positive Modulator of IL-6R and IL-6 Expression in the Subcutaneous Adipose Tissue: Significance for Metabolic Inflammation
Source: PLoS One. 2015 Jul 22;10(7):e0133494. doi: 10.1371/journal.pone.0133494 (PMC4511728; doi:10.1371/journal.pone.0133494)

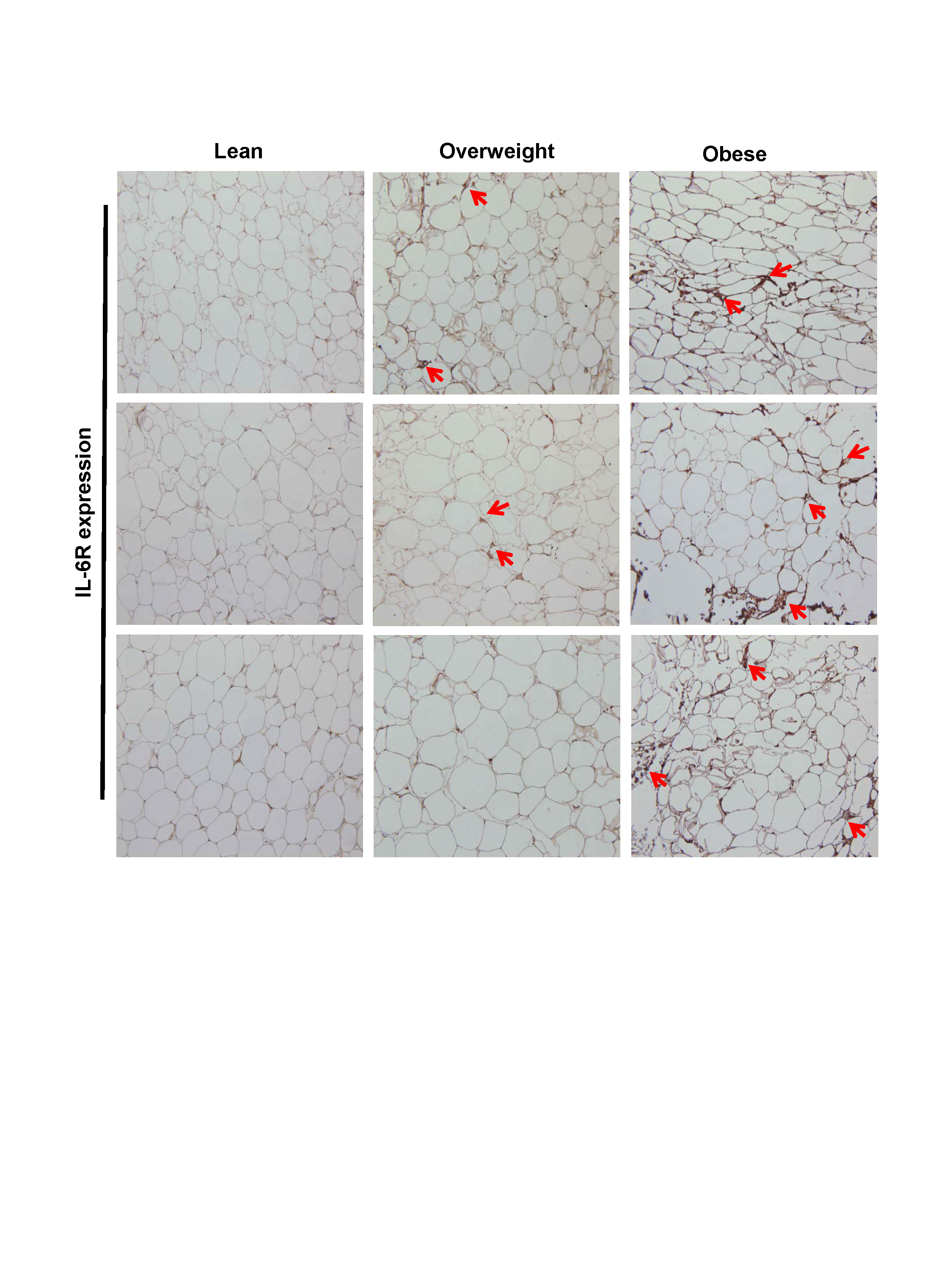

Supplement: S1 Fig — The subcutaneous adipose tissue samples were obtained by surgical biopsy from 10 lean/ overweigh (BMI = 20.285 to 29.456) and 10 obese (BMI = 31.752 to 38.218) non-diabetic individuals and protein expression (intensity) of IL-6R was measured by immunohistochemistry (IHC). The representative IHC photomicrographs (20× magnification) of IL-6R staining intensity (arrows) in the adipose tissue samples from lean, overweight, and obese individuals, 3 each, are shown. (TIFF) [file pone.0133494.s001.tiff]

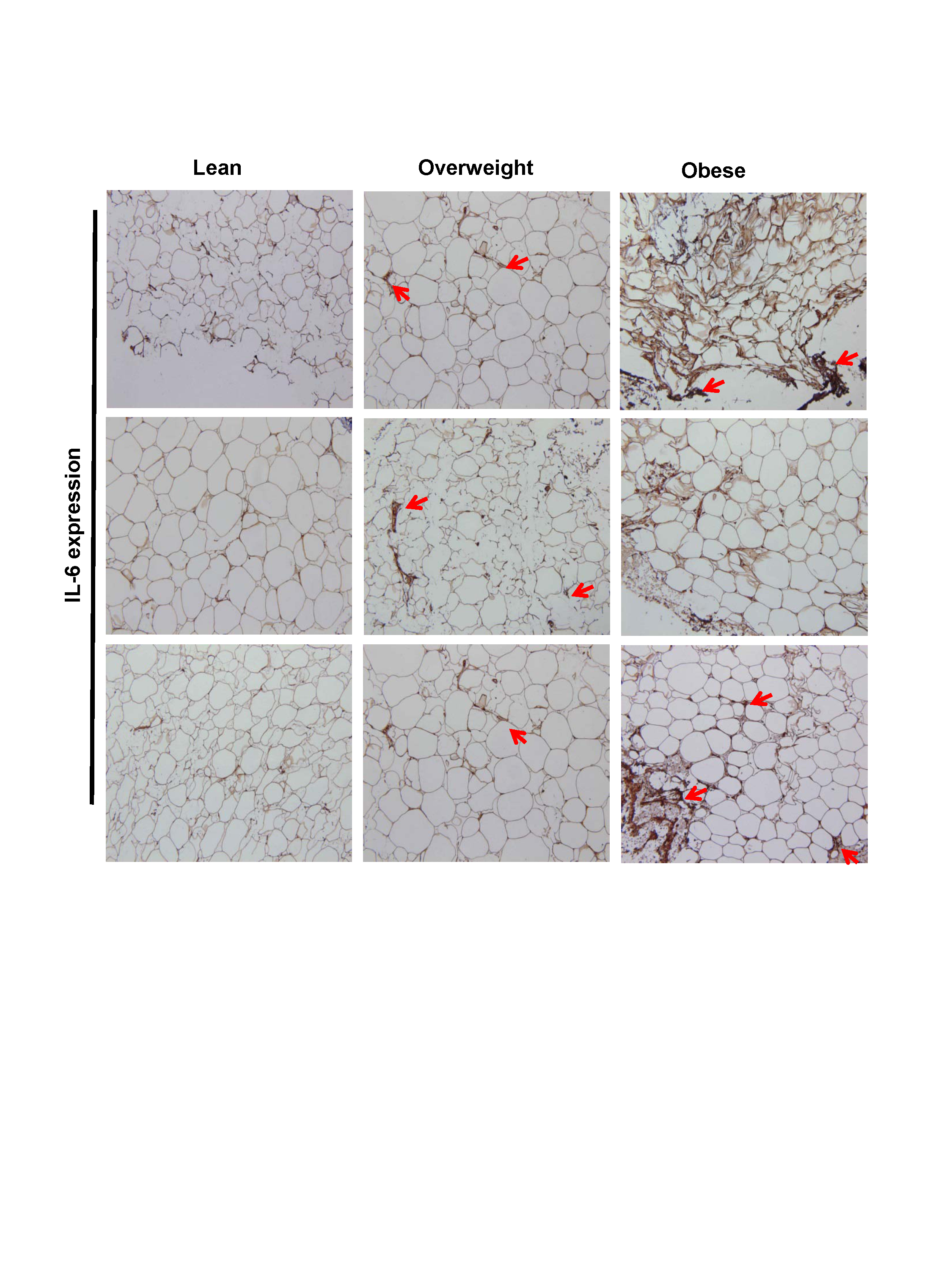

Supplement: S2 Fig — The subcutaneous adipose tissue samples were obtained by surgical biopsy from 10 lean/ overweigh (BMI = 20.285 to 29.456) and 10 obese (BMI = 31.752 to 38.218) non-diabetic individuals and protein expression (intensity) of IL-6 was measured by immunohistochemistry (IHC). The representative IHC photomicrographs (20× magnification) of IL-6 staining intensity (arrows) in the adipose tissue samples from lean, overweight, and obese individuals, 3 each, are shown. (TIFF) [file pone.0133494.s002.tiff]
